# The Internal Administration of Carbolic Acid in Pruritis and Prurigo

**Published:** 1871-05

**Authors:** 


The Internal Administration of Carbolic Acid in Pruritus and Prurigo.—Prof. Binz agrees with Hebra that pruritus and prurigo may be relieved by the internal administration of carbolic acid. The subject was lately discussed at Bonn, and a report given in the *Berliner Klinische Wochenschrift.* We should think the sulpho-carbolates, as given by Dr. Sansom, might be tried. We gave in our last number an epitome of Dr. Sansom's investigations, which have been detailed more fully in his paper read before the Medico-Chirurgical Society, and published in vol. lii of their "Transactions;" and also in his paper read before the British Medical Association at the last annual meeting, and published in a recent number of the journal.—*The Doctor.*

